# Time‐resolved dosimetry with pencil‐beam scanning for quality assurance/quality control in particle therapy

**DOI:** 10.1002/acm2.13397

**Published:** 2021-10-19

**Authors:** Soorim Han, Takuji Furukawa, Yousuke Hara, Shigekazu Fukuda

**Affiliations:** ^1^ Graduate School of Science Chiba University Chiba Japan; ^2^ QST Hospital National Institutes for Quantum and Radiological Science and Technology (QST) Chiba Japan; ^3^ National Institute of Radiological Science National Institutes for Quantum and Radiological Science and Technology (QST) Chiba Japan

## Abstract

This study aimed to measure dose in a scanning carbon beam‐irradiation field with high sampling rate that is sufficient for identifying spots and verifying the characteristics of the scanning beam that cannot generally be derived from the dose. To identify the spot, which is the smallest control unit of beam information during irradiation, effecting measurements with a sampling time of 10 μs or shorter is necessary. The provided dose within a specific time is referred to as time‐resolved dose (TRD). We designed a circuit for time‐resolved dosimetry using a fast‐data acquisition unit (SL1000, Yokogawa Electric Co.), which can measure 100 000 samples per second. Moreover, we used converters to enable a connection between an ionization chamber (IC) and the SL1000. TRD was measured successfully using point irradiation and two‐dimensional irradiation patterns in a scanned carbon beam. Based on the moving time of the spot obtained from the position monitor, the dose delivered to the IC from each spot position (spot dose) was interpreted. The spot dose, displacement of the chamber from the beam's center axis, and beam size were derived using TRD and position monitor outputs, which were measured concurrent with TRD. Spot dose up to a radius of 8 mm area from the IC's center were observed. Using the spot‐dose equations and simulation, we show that the spot dose of each position varies depending on the beam size and displacement of the IC's center from the beam's center axis. We devise an interpretation method for the characteristics that may apply to quality assurance, such as the verification of the trend for the beam axis and isocenter to coincide, as well as beam‐size verification.

## 1 INTRODUCTION

In recent years, rather than X‐ray radiotherapy, particle therapy, which uses proton, helium, or carbon ions, is becoming popular owing to its advantages, such as delivering a high‐dose concentration to a tumor target and low side‐effects on normal cells.^1^ In particular, three‐dimensional (3D) pencil‐beam scanning is an ideal irradiation technique for optimally utilizing the characteristics of high‐energy particle beams and providing adaptive dose delivery.^2^ For the Heavy‐Ion Medical Accelerator in Chiba (HIMAC) scanning irradiation method (employed at QST hospital), irradiation information (referred to as a “pattern”), which has 3D positions with the number of particles planned for delivery to these positions should be prepared, and the dose to each position should be accumulated on the basis of the available information. In the present study, the point that has such energy information, *x* and *y* positions, preset count (a target value that is proportional to a delivered particle on a spot position) is referred to as a “spot.”

**FIGURE 1:**
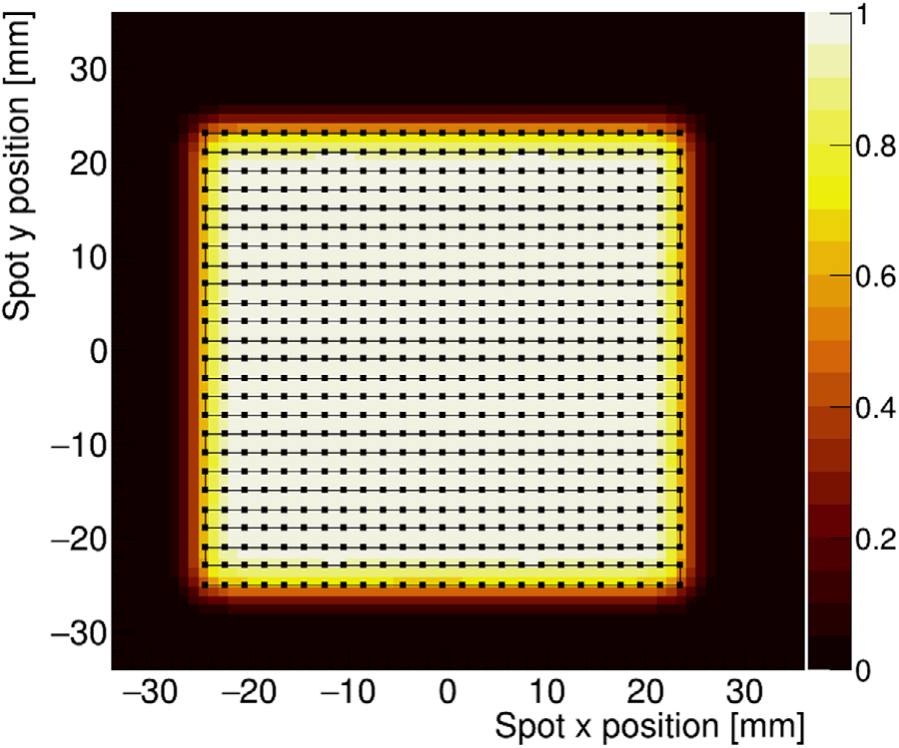
A simulated irradiation field measuring 48 × 48 mm^2^. The black squares are the irradiated spot positions, and the solid lines between the black squares are the irradiation route. In total, 625 spots were required to create this small two‐dimensional irradiation field of a 2‐mm spot distance

In advanced X‐ray radiotherapies, such as intensity modified radiotherapy (IMRT) and volumetric modulated arc therapy, several studies have been conducted to measure partial dose rather than an integrated dose to verify the dose at each control point (CP), which includes the gantry angle, the multileaf collimator aperture, and others. Because dose measurement is conducted over a period of seconds, this method is known as time‐resolved dosimetry (TRD).^3^ In the HIMAC system, the spots (not the CP) carry the most detailed information; therefore, the “spot” is a concept that is more closely related to the CP in IMRT. The spots are generated for all positions within a 3D field and, the number of spots is at least two digits larger than the number of CPs in X‐ray radiotherapy (Figure 1). Because the time between spots ranges from tens of microseconds to several milliseconds, measuring the dose at a microsecond interval is necessary to observe it from each spot (“spot dose”). Accordingly, we created an electric circuit that enabled us to perform high sampling‐rate dose measurements at each spot point around an ionization chamber (IC) placed in the isocenter.

We performed the first TRD system time evaluation using carbon ion pencil‐beam scanning to contemplate the applicability of the TRD measurement to quality assurance and quality control (QA/QC) with regard to the spot size of the beam and the coincidence of beam center and isocenter.

## 2 MATERIALS AND METHODS

### 2.1 The heavy‐ion medical accelerator in Chiba (HIMAC) of QST hospital

The therapeutic carbon beam of the HIMAC of the QST hospital is delivered through the 3D scanning of the pencil beam using a raster scanning method (lateral field) alongside full energy scanning (depth direction).^4^

Beam penetration depth is adjusted by changing the energy of the injected beam, which can be changed from 1.5 to 300 mm with using the particle energy of 55.6‐430 MeV/u.^5^ Lateral irradiation position is adjusted using X and Y scanning magnets for the *x* and *y* positions, respectively. The irradiation positions in the *x* and *y* directions are checked using a position monitor that has a 0.5‐mm resolution multiwire proportional counter. If a difference from the set irradiation position is detected, the irradiation position is brought closer to the planned position by applying the received feedback at the next CP. The maximum scanning speed reached 100 m/s for *x* and 50 m/s for *y*.

**FIGURE 2:**
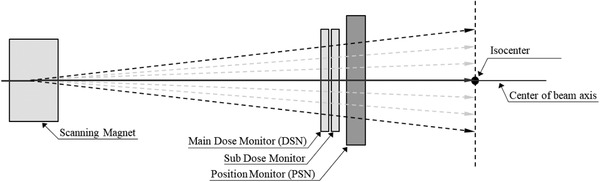
Irradiation monitoring system for the scanning beam port of the HIMAC. The monitoring system comprises two dose monitors (main and sub) and a position monitor. The monitors are sufficiently large to include the maximum scanning area. Ideally, the nonbended beam passes the isocenter of the treatment room

The irradiation dose is monitored through two, that is, main dose and subdose monitors. The dose monitors are ICs with thin windows, designed to monitor the number of incident particles passing through the monitor (Figure 2). The subdose monitor is used for the redundancy of the dose monitoring and this output is not used in this study. Ideally, the isocenter is at the center of the beam axis through which the nonbended beam passes. As the charged beam was scanned to a target position using the electromagnetic force of the scanning magnets, the three monitors provided sufficiently large coverage for the beam heading within the maximum field size. To precisely deliver the planned number of particles, the dose monitors continuously counted the number of incident particles; when the preset count was reached, the beam moved to the next spot.

### 2.2 Time‐resolved dosimetry

**FIGURE 3:**
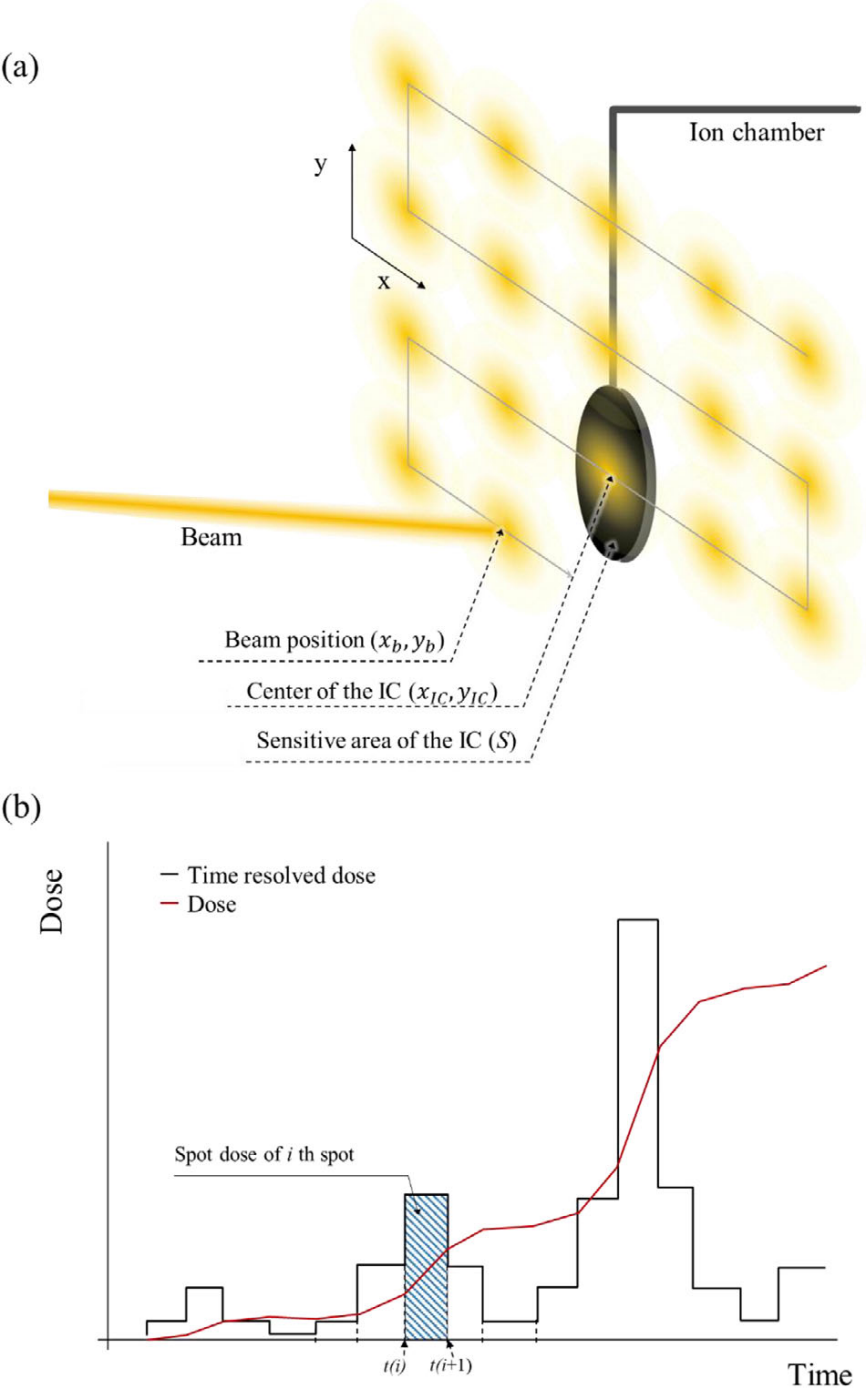
(a) Pencil‐beam scanning irradiation; (b) the concepts of TRD and integrated TRD. In (a), the blurry yellow spots indicate the position of spots when the beam is irradiated along the gray line; in (b), the measured TRD is indicated by the black histogram, spot dose is the TRD irradiated on the spot, and red line indicates the dose at the time

Figure 3 shows a schematic of the dose that each spot provided to the dosimeter. Consider a field in which a beam is projected onto the spots as shown by the blurry yellow spots in Figure 3a; the route of scanning is described as a gray line, and an IC is placed on a point in the field. Assuming the pencil beam has a Gaussian spread and the sampling time is sufficiently short, the signal read from the IC will match the histogram in Figure 3b.

If the route of an *x*‐line is close enough to deliver a measurable dose to the IC, one step‐like peak will be generated. Furthermore, for the *x*‐line created from the next *y*‐coordinate, another peak will be generated from a series of spots on the *x*‐axis. The integrated value of TRD between moving time to the *i*th spot (*t_s_* (*i*)) and that of the next spot (*t_s_* (*i* + 1)) can be defined as a spot dose of the *i*th spot (*D_spot_* (*i*)).

(1) $$D_{spot}\;\left(i\right)=\int_{t_{s}\left(i\right)}^{t_{s}\left(i+1\right)}TRD\left(t\right)\;dt\;.$$

Both the integrated value of TRD with time during whole irradiation time (*t_irr_*) and sum of spot dose will be the dose (D) at the position of the IC. This is described as the red line in Figure 3b.

(2) $$D\;=\int_{t_{irr}}TRD\left(t\right)\;dt\;=\sum_{i}^{All\;spots}D_{spot}\left(i\right).\;$$

Conversely, when the IC is placed at the isocenter (*x_IC_ *= 0, *y_IC_* = 0), TRD can theoretically be defined by Equation (3) as follows:

(3) $$\begin{array}{c} TRD\;\left(x_{b},\;y_{b},\;z,\;t\right)=\frac{dNP}{dt}\;\cdot \frac{dE}{dz}\left(z\right)\\ \cdot \int_{S}f\left(x_{b}-x,\;y_{b}-y,z\right)dxdy\\ \;=\frac{dNP}{dt}\;\cdot \frac{dE}{dz}\left(z\right)\cdot F\left(x_{b},\;y_{b},\;z\right) \end{array},$$

where *x_b_* and *y_b_* are the beam positions of the *x* and *y* directions, respectively; *dNP*/*dt* is the number of incident particles per time; *dE/dz* (*z*) is the unrestricted linear energy transfer (LET) on measuring depth *z* where the IC is placed; *f*(*x, y, z*) is a probability function of beam shape; and *F*(*x, y, z*) is a function that integrates function *f* with the sensitive area of the IC(*S*) (Figure 3a).

Using the Equations (1) and (3), the spot dose can be described in Equation (4) as follows:

(4) $$\begin{array}{c} D_{spot}\left(x_{b}\left(i\right),\;y_{b}\left(i\right),\;z\right)\;\;\\ =\int_{t_{s}\left(i\right)}^{t_{s}\left(i+1\right)}\frac{dNP}{dt}dt\;\cdot \frac{dE}{dz}\left(z\right)\cdot F\left(x_{b}\left(i\right),y_{b}\left(i\right),z\right)\\ =\;NP_{i}\cdot \frac{dE}{dz}\left(z\right)\cdot F\left(x_{b}\left(i\right),y_{b}\left(i\right),z\right) \end{array},$$

where the *NP_i_* is the number of incident particles in the *i*th spot, that is, the integrated value of *dNP*/*dt* in time from *t_s_* (*i*) to *t_s_* (*i* + 1).

When conducting the spot‐dose analysis, it was anticipated that the beam characteristics, such as beam shape, the positional relationship between the IC and beam positions, and the LET of the measuring depth, would be verified.

### 2.3 Circuit

**FIGURE 4:**
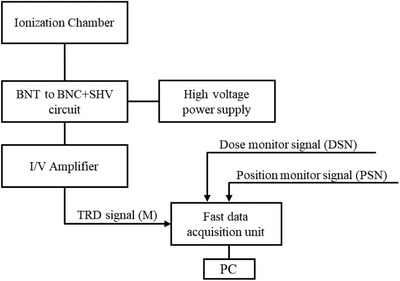
Diagram of the electric circuit used to measure the TRD. A self‐made conversion circuit (BNT cable to BNC cable plus SHV cable), current amplifier, and a high‐voltage supply were used to obtain the TRD signal. The fast‐data acquisition unit logged the TRD, dose monitor, and position monitor signals at the same time for the analysis of the spot‐dose

A diagram of the electric circuit used to measure the TRD is shown in Figure 4.

To log the output from an IC with a high sampling rate, an SL1000 (Yokogawa Electric Co., Tokyo) fast data acquisition unit, which can sample up to 1 000 000 samples per second, was used instead of a UNIDOS electrometer (PTW, Freiburg). The SL1000 supports only Bayonet Neill–Concelman (BNC) cable connection and input voltages from −10 to 10 V. Because the Advanced Markus IC (PTW, Freiburg) is a reference chamber for ion radiotherapy and has a simple shape for the radio‐sensitive area (a 5 mmϕ circle), this IC was employed for the first trial. The IC used in this study was a BNT type (triaxial BNC), which required the opposite of a BNC to connect to the SL1000. Because BNT cable supports signal, ground, and high voltage, when converting from BNT to BNC, a safe high voltage (SHV) cable was required for the applied voltage. Accordingly, a conversion circuit that converted BNT to BNC + SHV was created to change the types of the cable.

For the SL1000 to log, converting the current output to voltage and amplifying this value is necessary. Therefore, a Current Amplifier model 428 (Keithley Instruments, Ohio) was used. The gain of this amplifier converts the current into voltage and amplifies the value from $\times 10^{3}$ to $\times 10^{10}$. We set the gain of the current amplifier as $\times 10^{9}$, which is the highest gain that did not exceed the measurement limit of ±10 V.

The signals from the position (PSNX, PSNY) and main dose (DSN) monitor were also logged concurrently with TRD measurements. The time range from when the beam started to move to the *i*th spot (*t_s_* (*i*)) was obtained from the position monitor signal. As noted in section A, the DSN provided information proportional to the number of particles so that the value proportional to the *NP_i_* could be derived using the PSNX, PSNY, and DSN.

### 2.4 Irradiation

A water phantom was set with its surface placed at the isocenter. The *z*‐axis of the water phantom was set parallel to the beam axis (when it passes through the isocenter).

The beam intensity used for this measurement was 640 k spot counts per second, which is approximately 10^8^ particles per second. This beam intensity is the highest among the intensities commonly used for treatment.

We prepared two types of carbon‐beam irradiation patterns, that is, point irradiation affected by a pencil beam at 100 and 0 mm away from the IC center, and a two‐dimensional (2D) uniform field of 48 × 48 mm^2^. In the 2D pattern, we set the preset count of each spot as 640; in this way, the dwell time on a spot position became 1 ms. Moreover, spot counts were logged using the same sampling time as far TRD data, which enable us to analyze the influence of fluctuating the incident particles.

### 2.5 Calibration of time‐resolved dose

A calibration factor of IC for clinical use is recommended to use with an electrometer which is calibrated together with the IC. The Advanced Markus IC was calibrated for the use with the UNIDOS electrometer. However, because the calibration factor of the IC is not valid for use with the TRD circuit, obtaining the calibration factor using a calibration procedure is necessary.

Because the dose measured using UNIDOS is reliable, the calibration factor (*k_calib_*) can be obtained using a cross‐calibration method as shown in the Equation (5) below:

(5) $$k_{calib}=\frac{D_{ref,irr}}{\int_{t_{irr}}M\left(t\right)\;dt}.\;$$

Here, *D_ref,irr_* is the dose measured using the reference chamber set (Advanced Markus IC and UNIDOS) in an irradiation field, and *M*(*t*) is the value measured with the TRD circuit and same Advanced Markus IC in the same irradiation field, without changing any geometrical position in the reference measurement (only the connection of the IC and UNIDOS to the IC and TRC circuit was changed). As *M*(*t*) is the dose change over time, it must be integrated with time during the irradiation time(*t_irr_*).

The point irradiation pattern that delivers the dose in one spot for 5 s was used for the calibration to achieve a stable and sufficiently large dose.

### 2.6 Simulation of the time‐resolved dose

The simulation of TRD was performed with a self‐devised simulation code called “Spot dose” written in C++ and using the same 2D irradiation pattern. As in the 2D irradiation pattern, the spots were placed at 2‐mm intervals from −24 to 24 mm in *x* and *y* directions and moved in the same order as the real irradiation. The delivered dose while moving between the spots was ignored with the assumption that the scan speed was sufficiently fast. In this simulation, we assumed the number of particles within a given spot to be constant for all spots (*NP_i_* = const.). The sensitive volume of the Advanced Markus IC was implemented and the function of beam shape (*f*) was set as a 2D Gaussian shape for simplicity.

## 3 RESULTS

### 3.1 Irradiation outside and at the center of the ionization chamber

**FIGURE 5:**
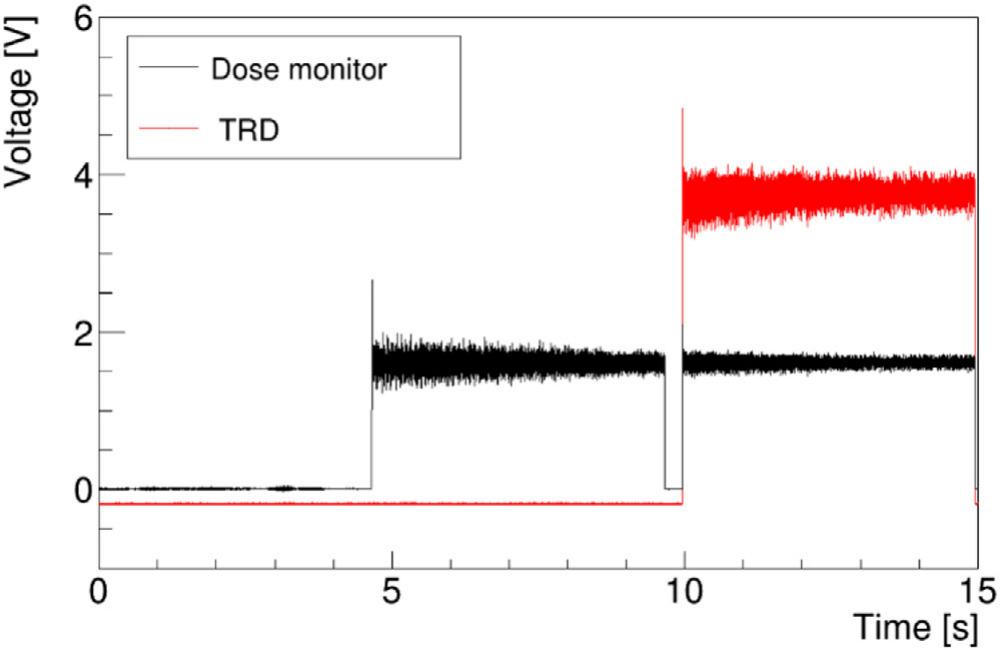
The point irradiation output. The red line shows the TRD output (*M*(*t*)), and the black line is the output of the DSN, which was measured at the same time as *M*(*t*). The irradiation at 100 and 0 mm (*x* and *y*, respectively) was started at approximately 5 s and ended at approximately 10 s. From 10 to 15 s, the beam was irradiated at the center of the IC^7^

Figure 5 shows the TRD spectrum when the beam was irradiated outside and at the center of the chamber. In the first period between 0 and 4 s, The beam was not irradiated. Between 5 and 10 s (the second period), the beam was irradiated at 100 and 0 mm (*x* and *y*, respectively). From 10 to 15 s (third period), the beam was irradiated at the center of the IC which was set on the isocenter. The noise average of the TRD was 0.18 V and had a standard deviation of 0.1 V (1 σ). Average noise changes due to repeated measurements and the beam being in an on/off were almost negligible. The magnitude of the signal detected when the beam passed through the center was 3.9 V on average, and the dose measured with UNIDOS in the same irradiation pattern was 3.08 Gy. The calibration factor derived from Equation (5) was 0.158 Gy/(V·s).

**FIGURE 6:**
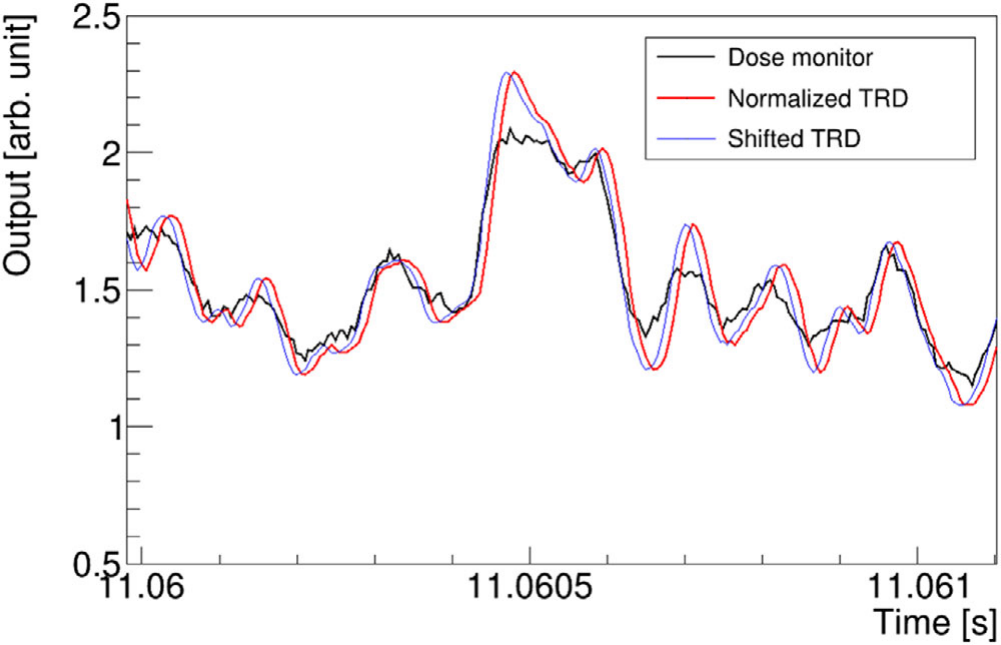
The synchronization of outputs. The black line is the output of the dose monitor, red is the normalized output of TRD for synchronizing the outputs, and blue is the synchronized data of TRD. The synchronization time was determined using the least‐squares method^7^

The DSN and TRD outputs show almost the same changes as in Figure 6. For a more accurate analysis in microseconds, the TRD data were synchronized to the dose monitor data using the least‐squares method (blue line in Figure 6). The synchronization time varied depending on the type of IC and gain of the current Amplifier. Using the Advanced Markus chamber and a gain of 10^9^, the synchronization time was 13 μs.

Within a large time scale (seconds), the dose delivered within 1 s was stable. However, when monitoring DSN with a short sampling time of 10 μs, the monitored number of particles per 10 μs exhibited significant fluctuation.

### 3.2 Two‐dimensional uniform field irradiation

**FIGURE 7:**
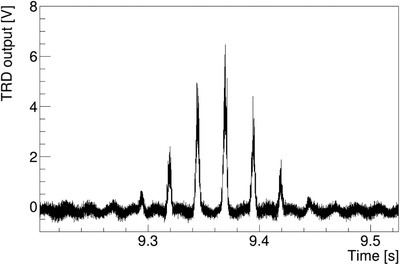
The TRD output over time during 2D irradiation. The raw TRD output measured with the sampling time of 10 μs of 2D uniform irradiation. Several peaks were found as expected in section B^7^

Figure 7 shows the TRD spectrum when the beam was irradiated using the 2D uniform field. The TRD spectrum confirmed the generation of several peaks. At first glance, more than six peaks were observed, and the TRD data (black line) showed significant fluctuation similar to single‐point irradiation. The time between the two peaks was 25 ms (as expected).

**FIGURE 8:**
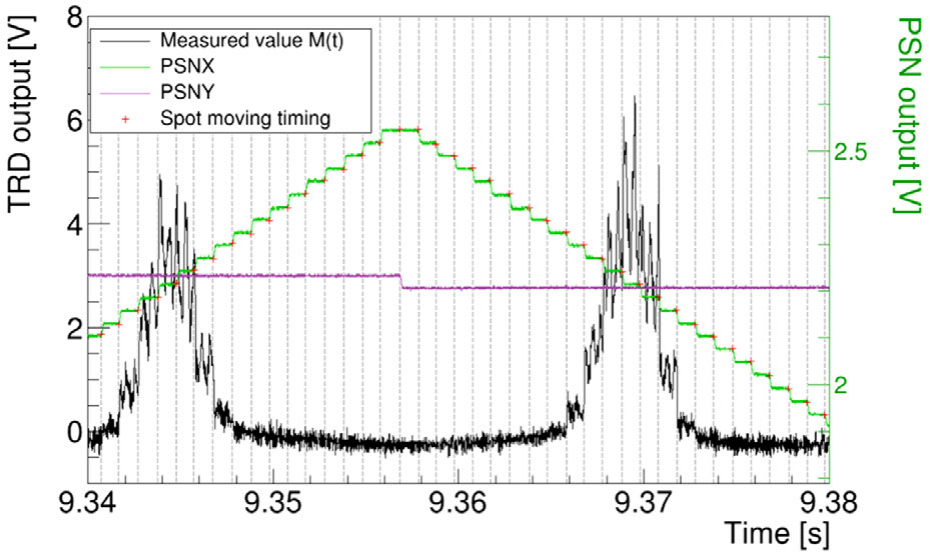
The TRD output for 2D uniform irradiation and spot moving timing derived from a position monitor. The black line is the magnified output of the TRD circuit for the two central peaks in Figure 7. The green and purple lines are the output of the position monitors for the *x* and *y* directions, respectively, which monitor the moving of the beam‐irradiation position. The red cross is the detected moving time derived using the calculation code developed in this study. The dashed line is an incidental line for reviewing the divided time intervals of the TRD output^7^

To obtain the spot dose for each spot, knowing where the beam is irradiated and when the position of the beam changes is necessary. We used position monitors to identify changes in beam spot timing. Figure 8 shows the output of the position monitors (PSNX, PSNY) and TRD output of the two largest peaks shown in Figure 7. The time change from one spot to the next (*t_s_* (*i*)) is noted with a red cross in Figure 8. The spot interval time was approximately 1 ms (as planned). The spot dose was obtained by integrating the measured values surrounded by the gray dashed lines in Figure 8, and multiplying them by a calibration coefficient (*k_calib_*).

### 3.3 Spot dose

**FIGURE 9:**
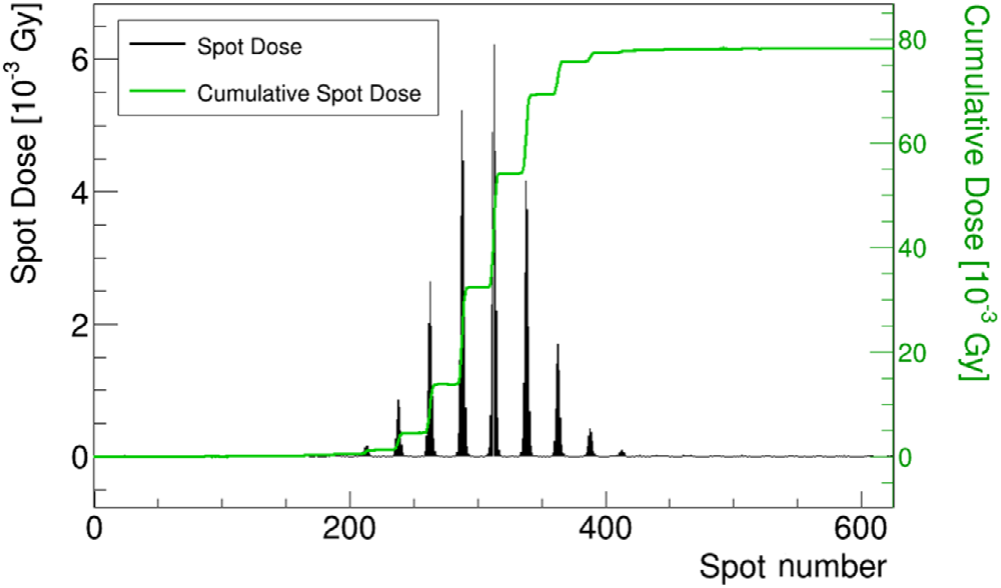
The spot dose and cumulative spot dose of 2D uniform irradiation

The spot dose (black bars) in Figure 9 is the integrated value of TRD over time for each spot (Equation 1). The 2D uniform irradiation pattern, introduced in section D, had 625 (25 × 25) spot points and we successfully obtained the same number of spots with the signal of the position monitor. From the highest peak rising at the center, it can be determined that the position of the IC did not deviate significantly in the direction of the *y*‐axis. The number of peaks was larger than those in the TRD‐time distribution, and the peaks could be more clearly identified. The spot‐dose noise was reduced to less than 1 × 10^−5^ Gy (1 σ).

The spot‐dose amount became 78.8 mGy, whereas the dose measured using the reference dosimetry (with UNIDOS) was 77.8 mGy.

**FIGURE 10:**
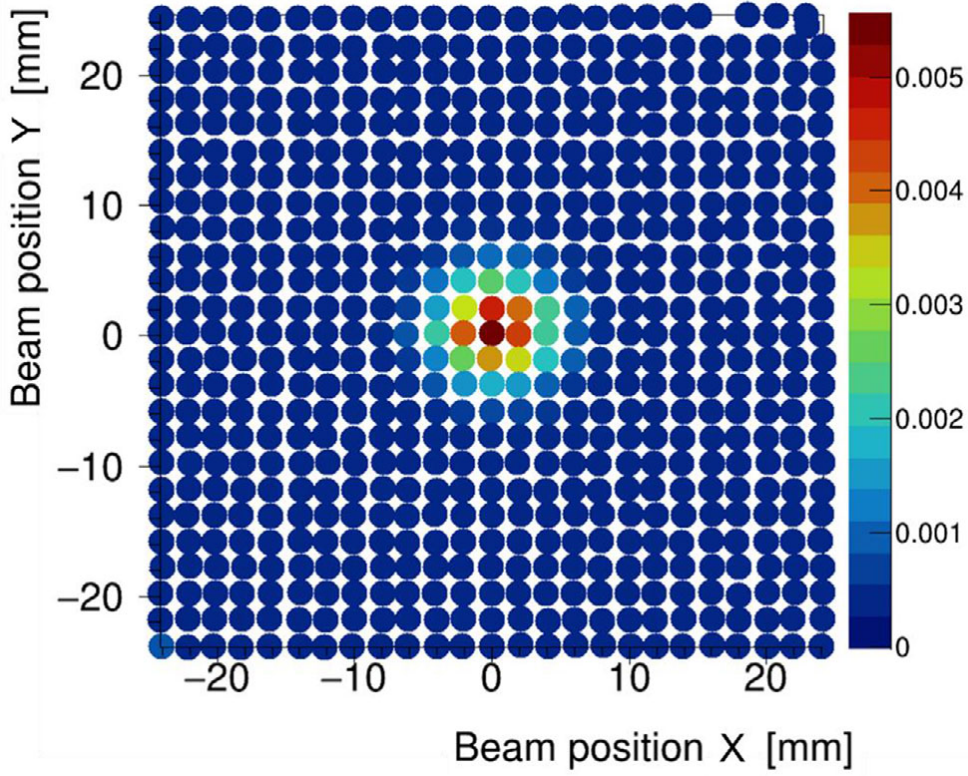
A 2D spot‐dose map plotted with information pertaining to the beam position for the PSNX, PSNY, and spot dose^7^

Combining the information provided by the PSNX, PSNY, and spot dose, we successfully created a 2D spot‐dose map (Figure 10). From the output of the IC (placed at the center), we derived the spot‐dose contribution of each spot to form a gross measurement point dose. This indicates one of the advantages of the TRD method, that is, obtaining the dose information from multiple beam positions with single irradiation in an IC.

**FIGURE 11:**
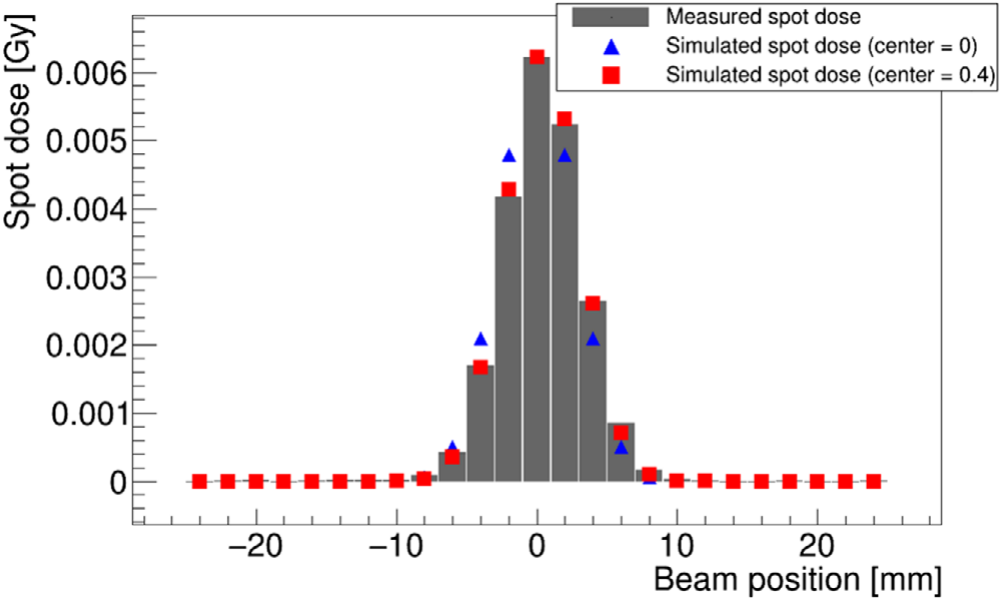
The spot dose of a centerline (*x* = 0). The gray bar chart represents a spot dose that was derived from 2D irradiation measurement. Both the blue triangles and red rectangles represent simulated spot‐dose data, but they have different measurement positions (0 and 0.4 mm, respectively)^7^

The gray bars in Figure 11 are the spot doses of the central line; the vertical profile of the center (*x* = 0) of Figure 10. We found significant data on the fourth bar from the center, which was on 8 mm away from the IC center. Similarly, the fourth peak from the center peak in Figure 9 represents the irradiated spots located at 8 mm from the center in the *y*‐direction of the dosimeter.

The blue triangle marker in Figure 11 is the data of the spot‐dose simulation in 2D plane with the condition of one Gaussian beam, which had a size of 2.3 mm (1 σ). This did not fit well with the measured spot dose. When the simulation was performed with an IC displacement position of 0.4 mm in the positive direction of *y*, however, the best agreement with the measured spot‐dose distribution (red square) was found. Similarly, we found the best agreement between horizontal line spot dose and simulated spot dose with a displacement of 0.1 mm in the *x*‐direction. When the same interpretation of the 10 repetitive 2D irradiation measurements was conducted, a displacement result was obtained within 0.07 mm (1 σ).

## 4 DISCUSSION

We attempted to confirm that the TRD analysis could be used in two verifications: (1) in 2D dose verification through the analysis of the spot dose and (2) in geometric verification through the interpretation of positional differences between the beam's center axis (Figure 2) and isocenter (where the IC is placed).

First, we attempted to verify the possibility of dose verification by interpreting the output of the position monitor to obtain the separation time between an individual spot and the TRD output. As shown in Figure 8, the timings of the movement of the spot (*t_s_*) were obtained from the position monitor output, and the number of movement times and number of spots in the simple 2D pattern were equal. The overall spot dose in one irradiation showed good agreement (1.2%) with the dose measured from the reference dose acquisition. This difference in dose could be reduced by improving noise reduction processing both during the measurement and in the analysis. Based on these results, it was confirmed that spot doses can be derived using the TRD analysis.

Based on the spot dose, observing which spot the dose had been derived from and how much weight it accumulated in the dosimeter was possible (Figure 10). Spot dose up to a radius of 8 mm area, which is wider than the radius of IC (2.5 mm), could be detected using the TRD system. This was sufficiently possible considering the size of the beams in the water phantom 8 mm is about 5.5 mm from the edge of IC, and 5.5 mm is about 2.4 sigma from the center of the beam. It means that on 8 mm, the spot dose of approximately 2% is delivered compared to the center of the beam. This 2% spot dose satisfied a sufficiently large value compared to noise so that the spot dose was identified in this area. This area is much smaller than the measurement area of a 2D array or film dosimetry; however, the point of interest regarding TRD measurement is different from these more general dosimetries. In the region around the IC, we were able to have the dose delivered from each spot, as well as the contribution of each spot to gross dose, which could not be derived from general dosimetry.

A favorable agreement existed between the spot‐dose distribution and cumulated dose with each simulation result (Figure 11) despite the beam shape in the simulation having a simple Gaussian shape (due to the large angle scattered particles, the beam shape of the carbon beam is typically expressed by the sum of three Gaussians^5^). The simulated data of the spot dose in Figure 8 were calculated under the condition that the position of the detector shifted by 0.1 and 0.4 mm in the *x* and *y* directions, respectively, from the beam center. It was clear that the displacement of the IC center from the isocenter, (*x_IC_*, *y_IC_*) caused a change in the distribution of function *F* as shown in Equation (6).

(6) $$D_{spot}\left(i,\;z\right)=NP_{i}\cdot \frac{dE}{dz}\left(z\right)\cdot F\left(x_{b}\left(i\right)-x_{IC},y_{b}\left(i\right)-y_{IC},z\right).$$

Using the above equation, we verified the difference between the beam center and isocenter where the IC was placed. Moreover, changes in *x* and *y* beam sizes were anticipated to change the shape of the spot‐dose distribution because the change in *f* (beam shape function; the original function of *F*) causes a change in the shape of *F*. This is applicable in mechanical QA/QC, for example, for the verification of the trend in which the beam axis and isocenter coincide,^6^ as well as in beam‐size verification.

The factors of uncertainty in spot dose are systematic uncertainty related to the IC and current amplifier, and rapid data acquisition unit, and random noise. The combined uncertainty of the three devices was 2.1% of the measured dose. The noise of spot dose was 10^−5^ Gy.

At the outset of this study, we attempted to obtain the delivered dose from each spot using the reverse‐calculation of the spot dose and distribution function *f* shown in Equation (7). The *IDD_spot_* is the integrated depth‐dose on depth which can be derived from *i*th spot. However, with the uncertainty calculated as shown in Equation (8), it was found that the evaluation of the *IDD_spot_* far from the center yielded significantly large uncertainty due to the noise of 10^−5^ Gy.

(7) $$IDD_{spot}\left(i\right)\;=\frac{D_{spot}\left(x_{b},\;y_{b},\;z\right)}{F\left(x_{b},\;y_{b},\;z\right)}\;=\;NP_{i}\cdot \frac{dE}{dz}\left(z\right),$$

(8) $$U_{IDD}^{2}={\left(\frac{0.021\cdot D_{spot}}{F\left(d_{x},\;d_{y},\;z\right)}\right)}^{2}\;+{\left(\frac{0.00001}{F\left(d_{x},\;d_{y},\;z\right)}\right)}^{2}$$

The right term in Equation (7) suggests an alternative method for obtaining the delivered dose of irradiation on each spot. In this study, we obtained information on the number of particles for each spot from DSN using the fast acquisition unit. Despite this method being unable to estimate the dose delivered while a spot was moving, the data could nonetheless help us verify the agreement between the planned number of particles and number of irradiated particles.

The 2D irradiation pattern had the same preset count for all spots for the sake of simplicity. Using the DSN signal, and geometrical analyses (displacement and beam size) are possible with other intricate patterns because the effect of the preset count on spot dose will be negated, thereby, leaving only linear energy and function *F*.

Three types of irradiation were included in this study, that is, spot irradiation of the center and outside the center with the TRD and UNIDOS systems each and 2D irradiation with the TRD system. Only 10 min was required for all irradiations and 5 min for changing UNIDOS to the TRD acquisition system.

## 5 CONCLUSIONS

In this study, TRD was conducted to measure the microdose delivered from a spot position to detector position. In the context of an irradiation system, this study reflects the first‐time application and evaluation of a TRD system using pencil scanning carbon beam.

A TRD of 100 kHz was measured using a fast‐data acquisition unit, which can measure at a high sampling rate and using an IC, which was employed as a reference dosimeter in treatment. The irradiation pattern at the IC's center and a uniform 2D preset count were used to simplify the analysis.

The moving times between spots obtained from the position monitor were used to interpret the spot dose. Using the position information of the beam provided by PSNX and PSNY, a 2D spot‐dose map was produced. This map represents the dose delivered to the IC from the beam irradiating at those positions, which was proportional to function *F*, not the dose delivered to those positions. The spot‐dose results showed good agreement with the simulation, which was optimized using the *x* and *y* positions of the IC and beam size. The optimized position of the IC indicated its displacement from the beam center.

The verification of the spot dose could be achieved even outside the IC's sensitive area. Although this area was much smaller than the measurement area of a 2D array, the TRD method provided the dose delivered from each spot, as well as the contribution to the dose of each spot around the IC, which could not be derived from a 2D array.

We initially tried to achieve the IDD or the number of incident particles in a spot delivered by the spot dose. However, the further the verification area from the IC, the worse the analysis accuracy of the IDD and the lower the number of the incident particles. Instead, we devised a method for dose verification by integrating the DSN measured during single spot dwell time to obtain the number of particles delivered. The comparison of the spot DSN and preset count will be performed in the subsequent study.

Using the characteristics of TRD, we confirmed the possibility of verifying the beam center axis and isocenter coinciding with the position of IC, spot size, and spot‐dose verification. Mechanical QA is, for the most part, not performed for the absolute estimation of each measurement, but for logging the trend and checking whether it includes significant differences from previous trends. In this sense, the method of verification using the beam center and beam size can perform sufficiently in the context of mechanical QA. Verification of spot dose deserves to be a new item of QA, because additional information in this regard will be useful, for example, establishing dose during the beam movements, at the same time as identifying additional contributions to microscopic doses. For 3D irradiations, it is expected to achieve additional characteristics which are changed with depth such as beam size and LET.

The study of the measurement system, irradiation method, method of analysis, and IC choice is not yet optimized. In this regard, it is certain that TRD measurement may be able to provide more information in a shorter time compared to general dose measurements.

## AUTHOR CONTRIBUTIONS

Takuji Furukawa conceived of the presented idea. Takuji Furukawa and Yousuke Hara verified the experimental methods.

Soorim Han, Shigekazu Fukuda, and Takuji Furukawa conceived and planned the experiments. Soorim Han and Shigekazu Fukuda carried out the experiment with support from Yousuke Hara. Shigekazu Fukuda helped to supervise the project.

Soorim Han and Shigekazu Fukuda contributed to the interpretation of the results. Soorim Han performed the analytic calculations and performed the numerical simulations.

Soorim Han took the lead in writing the manuscript. All authors provided a critical feedback and helped shape the research, analysis, and manuscript.

## CONFLICT OF INTEREST

The authors declare no conflict of interest.

## Data Availability

The data that support the findings of this study are openly available in figshare at https://doi.org/10.6084/m9.figshare.14480328, reference number 6.
